# Aortic valve repair versus biological replacement for aortic regurgitation with or without ascending aortic aneurysm: Heart Valve Society Aortic Valve Database Study

**DOI:** 10.1016/j.xjon.2026.102085

**Published:** 2026-08-26

**Authors:** Pichoy Danial, Adine R. de Keijzer, Johanna J.M. Takkenberg, Vincent Chauvette, Laurent de Kerchove, Jolanda Kluin, Claudia Romagnoni, Peter Verbrugghe, Pascal Leprince, Kevin M. Veen, Emmanuel Lansac

**Affiliations:** a Department of Cardiovascular and Thoracic Surgery, Pitié-Salpêtrière Hospital, Assistance Publique-Hôpitaux de Paris, Paris, France; b Department of Cardiology, Erasmus University Medical Centre, Rotterdam, The Netherlands; c Department of Cardiothoracic Surgery, Erasmus University Medical Centre, Rotterdam, The Netherlands; d Department of Cardiac Surgery, Montreal Heart Institute, Université de Montréal, Heart Institute, Montréal, Canada; e Department of Cardiothoracic and Vascular Surgery, Université Catholique de Louvain, Cliniques Universitaires Saint-Luc, Brussels, Belgium; f Department of Cardiothoracic Surgery, IRCCS Cà Granda Ospedale Maggiore Policlinico di Milano, Milano, Italy; g Department of Cardiovascular Sciences, Cardiac Surgery Unit, Leuven, Belgium

**Keywords:** aortic regurgitation, ascending aortic aneurysm, aortic valve repair, biological aortic valve replacement, long-term outcomes

## Abstract

**Objective:**

To compare the long-term outcomes of aortic valve repair (AVr) (REPAIR group) with biological aortic valve replacement (bAVR group).

**Methods:**

Adults who underwent REPAIR or bAVR for aortic regurgitation (AR) +/− ascending aortic aneurysm (AscAA), recorded in the Heart Valve Society database until April 2024, were included. Propensity score matching was used to adjust for confounders. Differences in long-term survival, major adverse valve-related events (MAVRE), and other valve-related events were assessed.

**Results:**

In total, 7318 patients were included (REPAIR n = 6773, bAVR n = 545). Propensity score matching resulted in 1006 REPAIR patients matched to 530 bAVR patients (median age: 67 years [interquartile range, 59.0-73.0], 22% female). In the matched cohort, early mortality (≤30 days) was comparable between REPAIR and bAVR patients (1.2% vs 2.1%, *P* = .070). At 7.5 years, overall matched survival was lower in the bAVR group compared to the REPAIR group (73.9% [95% CI, 66.5%-82.1%] vs 85.6% [95% CI, 82.3%-89.3%], *P* < .001), with a higher proportion of valve-related deaths (bAVR 4.2% vs REPAIR 1.2%, *P* < .001). At 7.5 years, patients with bAVR had significantly lower freedom from MAVRE (*P* < .001) and more infective endocarditis (*P* = .006), and severe valve dysfunction (*P* < .001) compared to patients with REPAIR. The incidences of aortic valve reintervention (*P* = .388) and pacemaker implantation (*P* = .671) were comparable.

**Conclusions:**

Among patients in whom repair was deemed feasible and surgery was performed at mainly expert centers, repair was associated with more favorable long-term outcomes, including improved survival, compared with bAVR in an older propensity-matched cohort. These findings support the consideration of AVr when technically feasible in patients with AR, with or without AscAA.

**Figure:**
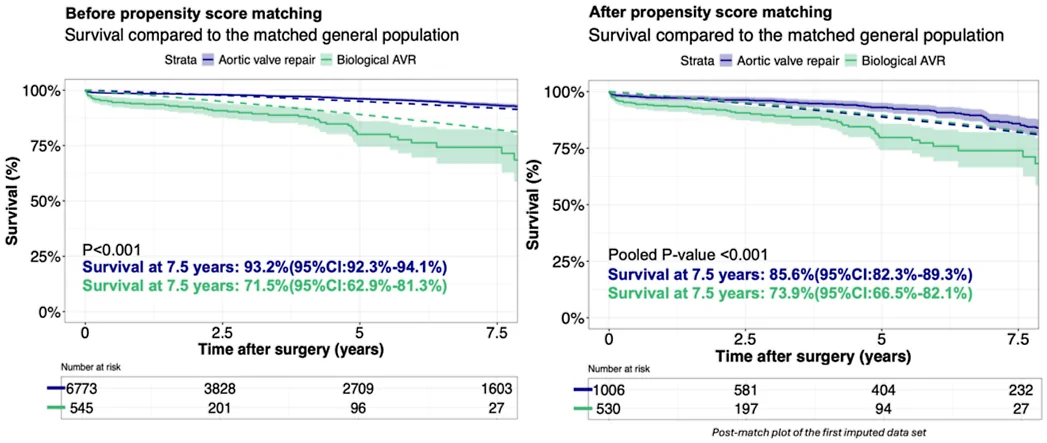
Kaplan-Meier survival after surgery versus matched population (repair vs bio AVR).

**Central Message**

Among patients in whom repair was deemed feasible and surgery was performed at expert centers, aortic valve repair was associated with superior long-term survival and fewer valve-related events compared with biological aortic valve replacement.

**Perspective**

In patients with aortic regurgitation with or without ascending aortic aneurysm in whom repair was deemed feasible and surgery was performed mainly by expert surgeons, aortic valve repair achieved survival comparable to that of the age- and sex-matched general population and was associated with lower valve-related morbidity than biological aortic valve replacement. For these patients, aortic valve repair should be considered the preferred initial strategy when technically feasible.

Aortic regurgitation (AR) arises from pathology of the aortic valve cusps and may occur in the presence or absence of an ascending aortic aneurysm (AscAA). Aortic valve replacement (AVR) is still used as the standard treatment in most patients with AR with a surgical indication,^1^ despite a significantly lower survival compared with the matched general population after surgery.^2^^,^^3^ Although guidelines indicate that mechanical AVR should be considered in patients under the age of 60 years,^4^ some advocate the use of a biological prosthesis in this younger patient population as well. This is partly driven by the possibility of less invasive transcatheter valve-in-valve reintervention procedures,^5^ lifestyle considerations, and a safer course during pregnancy.^6^ Therefore, biological AVR (bAVR) has gained broader use across all age groups over the past decade.

However, in recent years, aortic valve repair (AVr) has emerged as an alternative to AVR in patients with AR, showing good long-term survival comparable to the matched general population^7^, ^8^, ^9^, ^10^, ^11^ with low rates of valve-related events and good quality of life.^12^^,^^13^ AVr preserves the patients’ native aortic valve while avoiding the risks of anticoagulation and limiting other prosthesis-related events (eg, endocarditis).^2^^,^^3^^,^^14^ The Euro Heart Survey on Valvular Heart Disease found that two-thirds of the patients with AR had valves suitable for repair, but only 21.5% were actually treated with this option.^1^^,^^15^ The current guidelines state that AVr should be considered an alternative in selected patients, when performed by experienced surgeons (class IIa).^16^ In addition, the American College of Cardiology/American Heart Association guidelines recommend the consideration of AVr in selected patients with AR, particularly in experienced centers.^17^

Currently, there are limited data directly comparing outcomes of AVr with bAVR. Therefore, this multicenter study aims to compare long-term outcomes after AVr (REPAIR group) versus bAVR among adults undergoing surgery for AR with or without AscAA.

## Methods

### Study Population

The Heart Valve Society (HVS) Aortic Valve database was used to identify adult patients who had undergone REPAIR or bAVR. All patients with endocarditis, acute or chronic type A dissection, rheumatic disease, or aortic stenosis as a primary surgical indication were excluded. Ozaki procedures were excluded as well. Indications for intervention were based on the European Society of Cardiology/European Association of Cardio-Thoracic Surgery guidelines for the management of valvular heart disease.^4^

### Database and Ethical Approvals

The HVS Aortic Valve database is a longitudinal observational database enrolling patients with AscAA, AR, or aortic valve stenosis to investigate the natural history of aortic (valve) disease and long-term outcomes after surgical treatment. Patients were followed up ambispectively during outpatient clinical visits, where repeated echocardiographic measurements were collected. Most patients were included in the HVS database from 2013 onward. Details about the HVS Aortic Valve database were previously published.^18^ This study was approved by the scientific committee of the HVS Database, and local ethical approval was obtained at each participating center.

### Definitions

The REPAIR group included isolated valve repair ± tubular ascending aortic replacement or partial root replacement or valve-sparing root replacement (VSRR). VSRR included both the aortic valve reimplantation technique (David procedure) and the root remodeling technique (Yacoub procedure). AVR included isolated AVR with a biological aortic valve prosthesis ± tubular ascending aortic replacement or aortic root replacement (Bentall). Patients were assigned to the REPAIR group when a repair was initially performed, even if subsequently converted.

Early mortality was defined as 30-day mortality. Early aortic valve–related reintervention included in-hospital and/or ≤30-day reintervention. Reintervention was defined as either surgical or transcatheter intervention related to the aortic valve. Valve-related events were documented according to the Akins and colleagues’^19^ guidelines for mortality and morbidity after cardiac interventions. Valve-related mortality was defined as death due to valve dysfunction, valve-related complications, or sudden unexplained death. Postoperative moderate valve dysfunction was defined by either a mean aortic valve gradient between 20 and 40 mm Hg or the presence of moderate intraprosthetic AR - new or worsening compared with discharge baseline (≥ grade II). Postoperative severe valve dysfunction was defined as a mean aortic valve gradient of ≥40 mm Hg, or severe AR—new or worsening from the discharge baseline (≥ grade III)—or the occurrence of late aortic valve–related reoperation. A composite end point of major adverse valve-related event (MAVRE), including valve-related mortality (including sudden death), endocarditis, late aortic valve–related reintervention, late severe valve dysfunction, and pacemaker implantation <14 days after surgery, was also assessed.

### Statistical Analysis

Continuous data are presented as mean ± standard deviation for Gaussian distributions or median (interquartile range [IQR]) for non-Gaussian distributions. Categorical data are presented as frequencies (percentage). Comparisons among continuous variables were made with the *t* test, or the Mann-Whitney test, as appropriate. Comparisons of categorical variables were made with the χ^2^ test or with the Fisher exact test, as appropriate. Patients were stratified based on the type of surgery they underwent, REPAIR or bAVR.

Multiple imputation by the chained equations method was used to impute missing baseline variables.^20^ More details regarding the multiple imputation can be found in Online Data Supplement.

#### Confounder adjustment

Propensity score matching was performed to balance baseline differences in the pairwise comparison (REPAIR vs bAVR).^21^ A multivariable logistic regression model was developed to estimate the propensity scores. The type of surgery was the dependent variable (REPAIR/bAVR). A 2:1 matching strategy was applied with the caliper set at 0.2, using a nearest-neighbor matching algorithm without replacement. In the matching, isolated REPAIR was considered equivalent to isolated bAVR, partial root replacement and VSSR to the Bentall procedure, and REPAIR + tubular aortic replacement to bAVR + tubular aortic replacement. The models included age at surgery, sex, and concurrent surgeries as independent variables. Other baseline variables were added until all clinically relevant variables had a satisfactory balance with a standardized mean difference of <0.1. To gain insights into the average treatment effect, the inverse propensity treatment weighting (IPTW) method was applied as a sensitivity analysis.^22^ More details regarding the propensity score analysis and IPTW analysis can be found in Online Data Supplement.

#### Late outcomes

Survival and freedom from MAVRE were estimated by the Kaplan-Meier method and compared between groups using the log-rank test, both in pre- and postmatch settings. Methods of obtaining survival in the age- and sex-matched general population included a novel, patient-level matching strategy.^23^ All other outcomes/adverse events were presented as the cumulative incidence function and analyzed in the presence of death as competing risk using the Aalen-Johanssen estimator. The Gray test was used to compare groups in competitive risk analyses. Follow-up completeness was calculated using the Modified Clark C.^24^

All analyses were performed in R and Rstudio (Posit Software, version 4.5.1) using packages MatchIt, Survival, tidycmprsk, and WeightIt.

## Results

### Baseline Characteristics

In total, 7318 patients who underwent surgery met the inclusion criteria (REPAIR, n = 6773; bAVR, n = 545). The median year of surgery was 2015 (Online Data Supplement). Density plots of the propensity score in the unmatched and matched cohorts are shown in Online Data Supplement. Baseline characteristics of the unmatched and matched cohort are presented in Table 1. Online Data Supplement demonstrates unmatched and matched presurgery echocardiographic measurements, aortic valve morphology, and surgical details of the total cohort, further stratified by surgical technique.

**Table 1:** Baseline characteristics of the overall cohort and postmatch cohorts, first imputed data set is used

Variable	Before matching				After matching			
	Overall (n = 7318)	Aortic valve repair (n = 6773)	Biological valve replacement (n = 545)	*P* value	Overall (n = 1536)	Aortic valve repair (n = 1006)	Biological valve replacement (n = 530)	SMD
Age at surgery, y	54.0 (42.0, 65.0)	53.0 (41.0, 63.0)	68.0 (61.0, 73.0)	**<.001**	67.0 (59.0, 73.0)	67.0 (59.0, 73.0)	67.0 (61.0, 73.0)	−0.013
Female, n	1387 (19.0)	1264 (18.7)	123 (22.6)	**.026**	343 (22.3)	221 (22.0)	122 (23.0)	0.016
Height, cm	177.0 (170.0, 183.0)	177.0 (170.3, 183.0)	172.0 (165.0, 178.0)	**<.001**	172.0 (165.0, 178.0)	172.0 (165.0, 178.0)	172.0 (165.0, 178.0)	0.01
Weight, kg	82.0 (73.0, 93.0)	83.0 (73.0, 94.0)	79.0 (70.0, 89.5)	**<.001**	79.0 (70.0, 89.0)	79.0 (70.0, 89.0)	79.0 (69.0, 90.0)	0.037
Main reason for referral+, n								
Aortic regurgitation, n	4204 (57.4)	3885 (57.4)	319 (58.5)	.626	854 (55.6)	548 (54.5)	306 (57.7)	−0.061
Ascending aortic aneurysm, n	3114 (42.6)	2888 (42.6)	226 (41.5)	.626	682 (44.4)	458 (45.5)	224 (42.3)	−0.061
Previous surgery, n	385 (5.3)	348 (5.1)	37 (6.8)	.344	68 (4.4)	33 (3.3)	35 (6.6)	0.083
NYHA, n								
NYHA I, n	2361 (32.3)	2188 (32.3)	173 (31.7)	.470	546 (35.5)	380 (37.8)	166 (31.3)	0.039
NYHA II, n	2081 (28.4)	1840 (27.2)	241 (44.2)	**<.001**	620 (40.4)	386 (38.4)	234 (44.2)	0.039
NYHA III, n	1986 (27.1)	1889 (27.9)	97 (17.8)	**<.001**	262 (17.1)	166 (16.5)	96 (18.1)	0.039
NYHA IV, n	418 (5.7)	404 (6.0)	14 (2.6)	**.001**	46 (3.0)	32 (3.2)	14 (2.6)	0.039
Heart rhythm, n								
Sinus rhythm, n	6485 (88.6)	6055 (89.4)	430 (78.9)	**<.001**	1296 (84.4)	875 (87.0)	421 (79.4)	0.086
Atrial fibrillation, n	478 (6.5)	431 (6.4)	47 (8.6)	**.015**	136 (8.9)	91 (9.0)	45 (8.5)	0.086
Pacemaker, n	53 (0.7)	43 (0.6)	10 (1.8)	**.002**	22 (1.4)	12 (1.2)	10 (1.9)	0.086
Other, n	26 (0.4)	21 (0.3)	5 (0.9)	**.039**	6 (0.4)	1 (0.1)	5 (0.9)	0.086
LVEF, n								
Good >50%, n	5232 (71.5)	4835 (71.4)	397 (72.8)	**<.001**	1101 (71.7)	714 (71.0)	387 (73.0)	0.048
Moderate 31%-50%, n	978 (13.4)	861 (12.7)	117 (21.5)	**<.001**	282 (18.4)	170 (16.9)	112 (21.1)	0.048
Poor 21%-30%, n	79 (1.1)	63 (0.9)	16 (2.9)	**<.001**	35 (2.3)	19 (1.9)	16 (3.0)	0.048
Very poor <20%, n	52 (0.7)	47 (0.7)	5 (0.9)	.955	13 (0.8)	8 (0.8)	5 (0.9)	0.048
Hypertension, n	3196 (43.7)	2899 (42.8)	297 (54.5)	.905	726 (47.3)	435 (43.2)	291 (54.9)	0.016
COPD, n	281 (3.8)	241 (3.6)	40 (7.3)	.149	97 (6.3)	58 (5.8)	39 (7.4)	0.033
Diabetes, n								
IDDM, n	83 (1.1)	73 (1.1)	10 (1.8)	1.000	25 (1.6)	15 (1.5)	10 (1.9)	0.071
NIDDM, n	166 (2.3)	132 (1.9)	34 (6.2)	**.046**	62 (4.0)	29 (2.9)	33 (6.2)	0.071
Oral diabetes treatment, n	129 (1.8)	103 (1.5)	26 (4.8)	.772	47 (3.1)	22 (2.2)	25 (4.7)	0.045∗
Creatinine, mg/dL	1.0 (0.8, 1.0)	1.0 (0.8, 1.0)	1.0 (0.8, 1.1)	**<.001**	1.0 (0.8, 1.1)	1.0 (0.8, 1.1)	1.0 (0.8, 1.1)	0.091
Dialysis, n	23 (0.3)	19 (0.3)	4 (0.7)	.540	10 (0.7)	6 (0.6)	4 (0.8)	−0.011
Poor mobility, n	69 (0.9)	61 (0.9)	8 (1.5)	1.000	15 (1.0)	7 (0.7)	8 (1.5)	0.031
Extracardiac arteriopathy, n	128 (1.7)	112 (1.7)	16 (2.9)	.668	41 (2.7)	25 (2.5)	16 (3.0)	−0.062
Recent MI, n	56 (0.8)	53 (0.8)	3 (0.6)	.222	5 (0.3)	2 (0.2)	3 (0.6)	0.038
Critical state, n	27 (0.4)	24 (0.4)	3 (0.6)	1.000	5 (0.3)	2 (0.2)	3 (0.6)	−0.044
Pulmonary hypertension, n								
≤30 mm Hg, n	6410 (87.6)	6031 (89.0)	379 (69.5)	**<.001**	1202 (78.3)	826 (82.1)	376 (70.9)	0.018
31-55 mm Hg moderate, n	346 (4.7)	275 (4.1)	71 (13.0)	**<.001**	156 (10.2)	92 (9.1)	64 (12.1)	0.018
>55 mm Hg severe, n	116 (1.6)	105 (1.6)	11 (2.0)	.309	43 (2.8)	32 (3.2)	11 (2.1)	0.018
Connective tissue disease, n	409 (5.6)	398 (5.9)	11 (2.0)	**<.001**	28 (1.8)	17 (1.7)	11 (2.1)	0.012

Bold values indicate statistical significance. *P* ≤ .05 indicates statistical significance.

*NYHA*, New York Heart Association; *LVEF*, left ventricular ejection fraction; *COPD*, chronic obstructive pulmonary disease; *IDDM*, insulin-dependent diabetes mellitus; *NIDDM*, noninsulin-dependent diabetes mellitus; *MI*, myocardial infarction; *SMD*, standardized mean difference. + Patients could have both indications.

∗ SMD of unimputed data. Percentages were calculated including missing data.

Among the bAVR performed, the preoperative plan was to repair 13.2% (72/545) of cases, which was changed perioperatively after valve inspection without repair attempt. In the repair group, 0.6% (42/6773) cases were converted to AVR in a subsequent clamp session after the first repair attempt.

### Unmatched Outcomes

The median and mean follow-up of the total cohort were 3.2 (IQR, 0.4-7.0) years and 4.4 years, respectively. A total of 398 patients died (valve-related death, n = 154) during follow-up. In the bAVR group, 55 deaths (n = 22 [40%] valve-related) occurred and 343 deaths (n = 68 [19.8%] valve-related) in the REPAIR group.

Unadjusted prematching survival at 7.5 years after surgery was higher in the patients with REPAIR (93.2%, 95% CI, 92.3%-94.1%) compared with patients with bAVR (71.5%, 95% CI, 62.9%-81.3%, *P* < .001) (Figure 1, Table 2). Kaplan-Meier estimates of freedom from MAVRE in the unmatched cohorts are shown in Figure 2. Comparisons of other adverse (valve-related) events of the unmatched cohorts are presented in Online Data Supplement.

**Figure 1:**
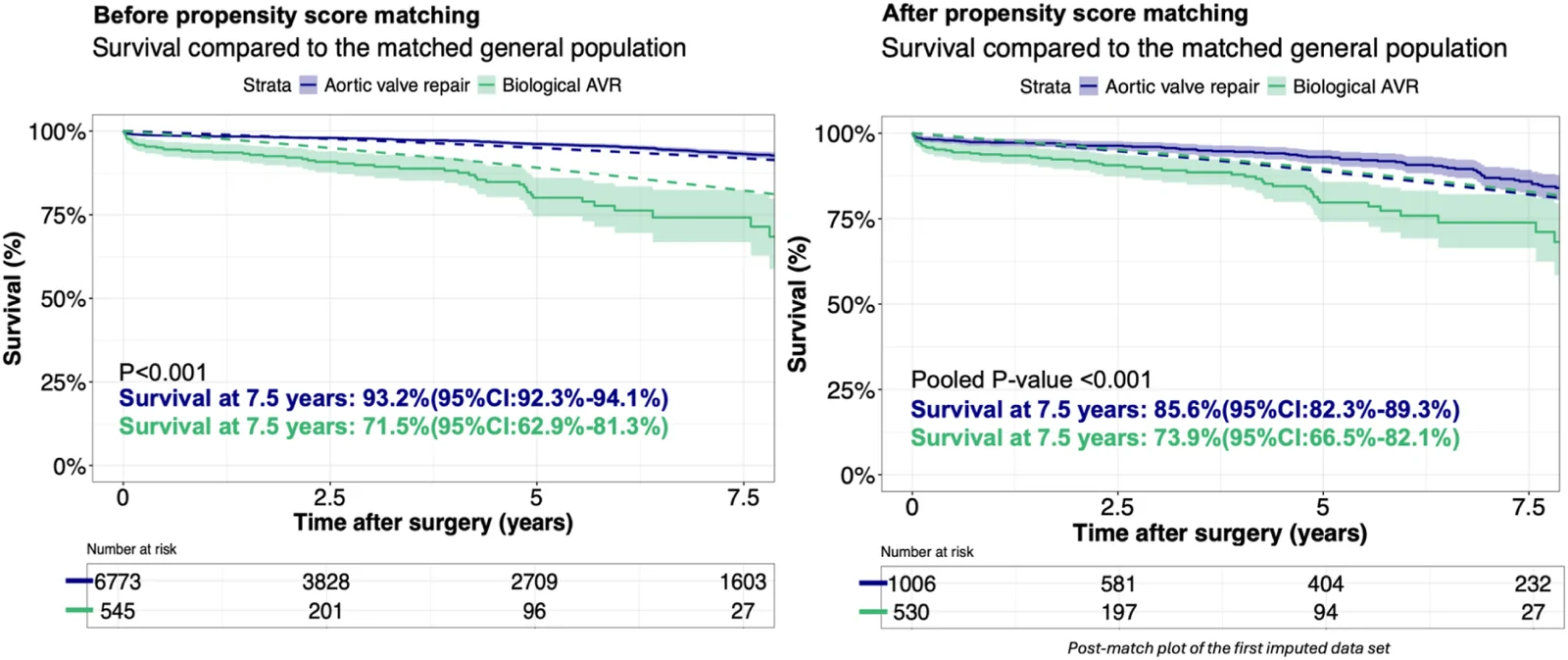
Kaplan-Meier estimates for overall survival after surgery compared to the matched general population for unmatched patients who had undergone aortic valve repair or biological aortic valve replacement (A) and matched patients who had undergone aortic valve repair or biological aortic valve replacement (B). The dotted line represents the age- and sex-matched general population. Figure contains the 95% CI.

**Table 2:** Unmatched and matched survival and other late time-to-event outcomes for patients who had undergone aortic valve repair or biological aortic valve replacement, including log-rank test or Gray test, as appropriate

Outcomes	Before matching					After matching∗				
	Aortic valve repair 5-y	Aortic valve repair 7.5-y	Biological valve replacement 5-y	Biological valve replacement 7.5-y	*P* value	Aortic valve repair 5-y	Aortic valve repair 7.5-y	Biological valve replacement 5-y	Biological valve replacement 7.5-y	Pooled *P* value
Survival	96.1% (95.5%-96.7%)	93.2% (92.3%-94.1%)	80.0% (74.5%-86.0%)	71.5% (62.9%-81.3%)	**<.001**	93.0% (91.0%-95.1%)	85.6% (82.3%-89.3%)	79.6% (74.1%-85.7%)	73.9% (66.5%-82.1%)	**<.001**
Freedom from MAVRE	90.7% (89.8%-91.5%)	86.3% (85.1%-87.6%)	77.8% (72.6%-83.6%)	67.9% (59.4%-77.6%)	**<.001**	94.0% (92.1%-96.0%)	90.2% (87.3%-93.1%)	82.5% (77.7%-87.6%)	72.5% (63.5%-82.8%)	**<.001**
Late aortic valve reoperation	5.5% (4.8%-6.2%)	8.2% (7.3%-9.2%)	3.4% (1.5%-6.6%)	3.4% (1.5%-6.6%)	**.020**	3.6% (2.3%-5.3%)	6.2% (4.2%-8.7%)	3.5% (1.5%-6.7%)	3.5% (1.5%-6.7%)	.388
Other cardiac reoperation	1.7% (1.3%-2.1%)	1.9% (1.5%-2.4%)	4.5% (2.4%-7.8%)	4.5% (2.4%-7.8%)	**.011**	1.9% (1.1%-3.1%)	2.2% (1.2%-3.6%)	4.3% (2.1%-7.5%)	4.3% (2.1%-7.5%)	.101
Endocarditis	0.5% (0.3%-0.8%)	0.6% (0.4%-0.9%)	2.4% (0.9%-5.3%)	3.6% (1.3%-7.9%)	**<.001**	0.6% (0.2%-1.6%)	0.6% (0.2%-1.6%)	2.4% (0.9%-5.4%)	3.7% (1.3%-7.9%)	**.006**
Aortic valve thrombosis	0.1% (0.1%-0.2%)	0.1% (0.1%-0.2%)	0.4% (0.04%-2.0%)	0.4% (0.04%-2.0%)	.400	0.3% (0.1%-0.9%)	0.3% (0.1%-0.9%)	0.4% (0.04%-2.0%)	0.4% (0.04%-2.0%)	.822
Aortic complication	0.7% (0.5%-1.1%)	1.0% (0.7%-1.4%)	0.7% (0.2%-2.5%)	0.7% (0.2%-2.5%)	>.900	1.6% (0.8%-2.8%)	1.6% (0.8%-2.8%)	0.8% (0.2%-2.5%)	0.8% (0.2%-2.5%)	.713
Pacemaker implantation	1.7% (1.3%-2.2%)	2.4% (1.9%-2.9%)	4.4% (1.6%-6.6%)	6.0% (2.7%-11.0%)	**.011**	3.4% (2.2%-5.1%)	4.5% (3.0%-6.6%)	3.5% (1.6%-6.7%)	6.1% (2.7%-11.0%)	.671
Moderate valve dysfunction	23.0% (22.0%-25.0%)	31.0% (30.0%-33.0%)	14.0% (9.5%-19.05)	15.0% (10.0%-21.0%)	**<.001**	27.0% (24.0%-31.0%)	34.0% (30.0%-38.0%)	14.0% (9.7%-20.0%)	16.0% (10.0%-22.0%)	**<.001**
Severe valve dysfunction	5.9% (5.2%-6.7%)	8.7% (7.7%-9.7%)	10.0% (7.3%-14.0%)	10.0% (7.3%-14.0%)	**.002**	3.8% (2.5%-5.6%)	6.2% (4.2%-8.7%)	10.0% (7.2%-14.0%)	10.0% (7.2%-14.0%)	**<.001**

Pooled *P* value represents the pooled *P* value of all 5 imputed data sets used for matching. Bold values indicate statistical significance. *P* ≤ .05 indicates statistical significance. *MAVRE*, Major adverse valve-related event.

∗ Estimates of first imputed data set.

**Figure 2:**
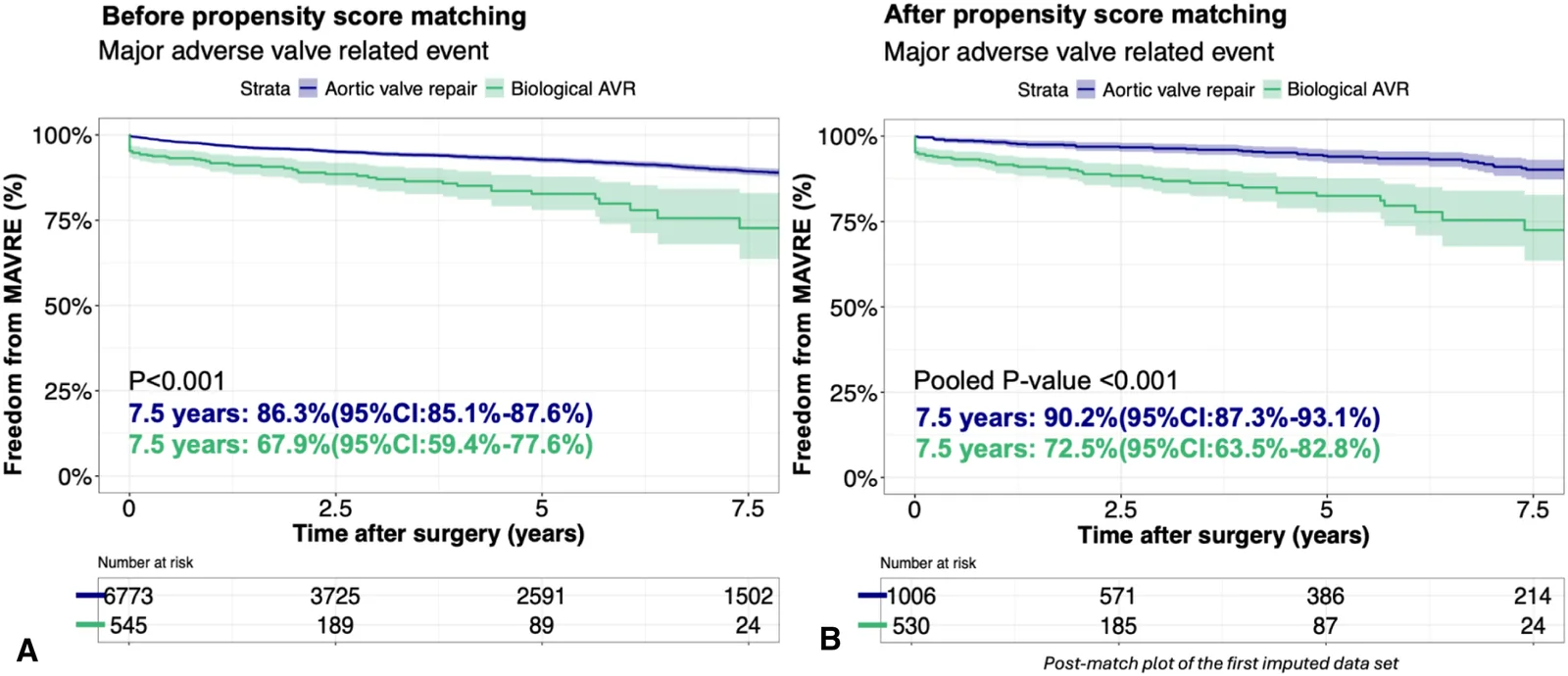
Unmatched and matched Kaplan-Meier estimates for major adverse valve related events for patients who had undergone aortic valve repair (A) or biological aortic valve replacement (B). Figure contains the 95% CI.

### Matched Outcomes

Propensity score matching resulted in 1006 patients who underwent REPAIR matched with 530 patients who underwent bAVR (Table 1). The overall early mortality was 1.5% and was comparable between the bAVR group (2.1%) and the REPAIR group (1.2%) (*P* = .070). At 7.5 years, overall matched postoperative survival was lower in the bAVR group (73.9%, 95% CI, 66.5%-82.1%) compared with the REPAIR group (85.6%, 95% CI, 82.3%-89.3%, pooled *P* < .001) (Figure 1). The proportion of valve-related death during follow-up was significantly larger in the bAVR group (4.2%) compared with the REPAIR group (1.2%, *P* < .001).

#### Reintervention

Early reintervention occurred in 13 patients (12 [1.2%] REPAIR, 1 [0.2%] bAVR, *P* = .142). Late reintervention at 7.5 years postoperatively was comparable between the bAVR group and the REPAIR group (3.5%, 95% CI, 1.5%-6.7% vs 6.2%, 95% CI, 4.2%-8.7%; pooled *P* = .388) (Online Data Supplement). In total, 2 transcatheter reinterventions were performed both in the bAVR group; one late reintervention using TAVR and one early reintervention with intraventricular device implantation for paravalvular leakage.

#### Morbidity

At 7.5 years after surgery, patients in the bAVR group had lower freedom from MAVRE compared to patients in the REPAIR group (72.5%, 95% CI, 63.5%-82.8% vs 90.2%, 95% CI, 87.3%-93.1%, pooled *P* < .001) (Figure 2, Table 2). The cumulative incidence of infective endocarditis was significantly higher in the bAVR group (3.7%, 95% CI, 1.3%-7.9%) compared to the REPAIR group (0.6%, 95% CI, 0.2%-1.6%) (pooled *P* = .006) at 7.5 years (Table 2, Online Data Supplement). Additionally, the bAVR group had a higher cumulative incidence of severe valve dysfunction (including reintervention) at 7.5 years after surgery compared with the REPAIR group (10.0%, 95% CI, 7.2%-14.0% vs 6.2%, 95% CI, 4.2%-8.7%; pooled *P* < .001), while cumulative incidence of moderate valve dysfunction was higher in REPAIR group (34.0%, 95% CI, 30.0%-38.0% vs 16.0%, 95% CI, 10.0%-22.0%; pooled *P* < .001) (Table 2, Online Data Supplement).

The cumulative incidences of aortic complications (Table 2, Online Data Supplement; pooled *P* = .713), other cardiac reintervention (Table 2, Online Data Supplement; pooled *P* = .101), pacemaker implantation (Table 2, Online Data Supplement; pooled *P* = .671), and aortic valve thrombosis (Table 2; pooled *P* = .822) at 7.5 years after surgery were comparable between the bAVR group and the REPAIR group.

### Sensitivity Analysis

After performing IPTW analysis, the hazard for mortality was significantly higher in the bAVR group compared with the REPAIR group (hazard ratio [HR], 2.2; 95% CI, 1.3-3.7; *P* = .002) (Online Data Supplement). Similar results were found for the hazard for MAVRE (HR, 2.8; 95% CI, 1.6-4.9; *P* < .001) and infective endocarditis (HR, 8.5; 95% CI, 2.2-33.0; *P* = .002), both significantly higher in the bAVR group compared with the REPAIR group. The IPTW weighted Cox proportional hazards models for other events can be found in Online Data Supplement.

## Discussion

The current study is, to our knowledge, the first comparison between REPAIR and bAVR based on a large, high-volume multicenter cohort of patients undergoing surgery for AR, encompassing the full clinical spectrum, including patients with or without AscAA, Marfan syndrome, and bicuspid or tricuspid aortic valves, with more than 95% of the patients presenting with at least mild AR. In the current matched cohort, patients who underwent REPAIR and were found to be repairable at primarily expert centers showed better long-term survival, a lower proportion of valve-related death, and a reduced incidence of MAVRE, infective endocarditis, and severe valve dysfunction but more moderate valve dysfunction compared with patients undergoing bAVR. The REPAIR group showed comparable survival with its age- and sex-matched general population, unlike the bAVR group. Occurrence of late aortic valve reintervention was comparable between groups. However, the study contributes meaningful, albeit not definitive, evidence to inform the ongoing discussion regarding the role of valve repair versus bAVR for patients with AR with or without AscAA; however, these results should be interpreted with caution, as center and surgeon preferences in treatment allocation and expertise might have played a role.

### Long-Term Postoperative Outcomes and Mortality

In the current study, postoperative survival at 7.5 years in the matched bAVR group was 74% (median age, 67) and not comparable with the age- and sex-matched general population. An impaired life expectancy compared with the matched general population has been previously described in patients who underwent bAVR,^2^^,^^3^ and may be attributable to higher observed rates of MAVRE and endocarditis, as also reflected by the significantly greater incidence of valve-related mortality in this group. However, besides higher valve-related mortality, excess mortality not directly related to valve-related events is also observed to be higher in bAVR patients compared with, for example, the Ross procedure.^2^^,^^25^ In contrast, other studies reporting outcomes after AVr, all single-center, have reported AVr to have a comparable survival compared to the matched general population and few valve-related events.^7^, ^8^, ^9^, ^10^, ^11^ The current study also showed comparable survival after AVr compared with the age- and sex-matched general population, this is found for the first time in a multicenter setting. When comparing AVr and bAVR, other studies also reported an impaired survival for bAVR patients compared with AVr patients.^26^^,^^27^ A potential explanation might be the less favorable hemodynamics of bioprostheses^28^; bioprosthetic valves introduce a more rigid annular structure and are associated with higher transvalvular gradients.^2^^,^^12^ It has also been shown that patients undergoing repair tend to experience good postoperative left ventricular remodeling and function.^29^^,^^30^ This may result in lower rates of subclinical fibrosis and preserved systolic performance in AVr patients.

The incidence of moderate valve dysfunction was relatively high in the REPAIR group at mid-term follow-up with the steepest increase in incidence within the first year postoperatively. This did, however, not appear to translate to clinically significant valve failure, as rates of severe dysfunction and reintervention remained low. This finding is consistent with the recent study by Zito and colleagues, which reported the occurrence of grade 2 AR, while moderate-to-severe AR remained rare up to 15 years after repair. bAVR exhibited higher rates of severe valve dysfunction; however, this did not translate into higher reintervention rates. Notably, prior studies showed that reinterventions after bAVR typically occur beyond 7.5 years.^2^ Given that bAVR has only been included in the HVS database in recent years, such late reinterventions may not yet be captured.

### Patient Selection and Surgical Management

There is still no ideal aortic valve prosthesis for patients with aortic valve disease. Of course, patient preferences and lifestyle considerations remain important aspects when discussing surgical treatment options for aortic valve surgery. As mentioned before, although still the standard treatment strategy, every aortic valve prosthesis has significant drawbacks.^2^^,^^3^ Aortic valve surgeries leaving a living valve as a result (eg, the Ross procedure and AVr) are the only aortic valve surgeries showing comparable survival with the age- and sex-matched general population and with relatively low valve-related events,^11^^,^^31^ even in patients with bicuspid aortic valves.^8^^,^^10^ AVr is relatively underutilized for treating AR since only 21.5% of aortic valves are repaired while 80% of dystrophic AR cases are repairable.^1^^,^^10^ In the meantime, bAVR is increasingly favored over mechanical valve implantation in the case of AR, including among patients younger than 60 years. This reflects lifestyle considerations (eg, sports and physical activity), the potential for future valve-in-valve transcatheter implantation,^1^^,^^32^ and the needs of women contemplating pregnancy.^6^ Like mitral repair, AVr is undergoing major changes following the advent of standardized repair techniques a decade ago.^33^, ^34^, ^35^, ^36^ However, usually, the choice of AVr is among others dependent on aortic valve morphology, patient age, and surgeon’s and center’s preference. There is a large variability between surgeons and centers, with specialized centers being more prone to perform repair in more complex cases. In the current cohort, about 13% of the patients who were planned for AVr received a bAVR after inspection of the cusps. However, cusp quality, although partly corrected for in the current matching strategies, may significantly influence treatment allocation and valve durability after repair. Adequate cusp tissue, characterized by sufficient geometric height, limited calcification, and absence of significant retraction, is essential for achieving a durable repair, whereas severely degenerated cusps often necessitate replacement.^37^^,^^38^ Importantly, while impaired cusp quality is associated with reduced feasibility and potentially lower durability of repair, the need for additional cusp repair techniques in themselves does not appear to adversely affect long-term outcomes when a competent valve configuration is achieved.^39^ This suggests that cusp quality primarily acts as a determinant of treatment allocation and technical complexity of repair, rather than directly influencing postoperative outcomes in bAVR, where standardized prosthetic performance may mitigate these anatomical differences.

Finally, the age of the matched sample was relatively high, inherent to the characteristics of the patients receiving bAVR. This raises questions about generalizability to younger patients. The observed benefit of repair may represent a conservative estimate, as greater advantages may be expected in younger patients. The use of biological prostheses has expanded progressively to younger patients over the past decade, driven by lifestyle considerations and the availability of valve-in-valve options. It is important to note that the emerging trend of bioprosthesis use in younger patient populations may not yet be sufficiently captured in the current data set. Further studies are therefore needed to evaluate outcomes of REPAIR versus bAVR in younger patient cohorts as this trend evolves. In younger patients, mechanical AVR is currently preferred over bAVR; however, it was not included in the current study. However, when comparing AVr to mechanical AVR, previous studies showed that mechanical AVR had impaired postoperative survival and, in some cohorts, also a greater number of valve-related events.^15^^,^^40^ Dedicated comparative studies are needed to continue evaluating long-term outcomes between repair and mechanical AVR.

### Limitations

The main limitation of this study is its observational design. The HVS aortic valve database only recently began including AVRs, particularly biological valve prostheses. Therefore, the follow-up time for the valve replacement groups was shorter than that for the AVr group. Finally, selection bias remains inherent to the observational design of this study. In clinical practice, AVr is preferentially performed in patients of younger age and more favorable valve anatomy (pliable cusps and limited calcification), whereas biological valve replacement may be selected in patients with less suitable anatomy or more advanced disease. This choice in treatment allocation is also dependent on the surgeon and the center. Most centers participating in the HVS aortic valve database are experienced centers for AVr where sometimes the procedures are performed by a single surgeon. Therefore, these findings likely reflect the results achievable in specialized centers with established expertise in AVr, and thus, the generalizability of this study must be confirmed in other studies. While propensity score matching and IPTW reduce the effect of confounders, they do not eliminate them completely and do not account for possible unmeasured confounders. Although our propensity score matching achieved a high level of balance across key variables, center and surgeon variability cannot be fully excluded. Additionally, the lack of age-stratified analyses represents an important limitation.

## Conclusions

In our propensity-matched and IPTW-adjusted cohort of patients considered suitable for repair, with surgeries predominantly performed at expert centers, AVr was associated with improved long-term survival, a lower proportion of valve-related death, and a reduced incidence of MAVRE and infective endocarditis, compared with those undergoing bAVR. Additionally, AVr was associated with better preservation of valve function and there were no differences in reintervention compared with bAVR. The study contributes meaningful, albeit not definitive, evidence to inform the ongoing discussion regarding the role of valve repair versus bAVR for patients with AR with or without AscAA who have repairable valves.

## Conflict of Interest Statement

E. Lansac reported a consultancy relationship with Coroneo. P. Verbrugghe reported to be a consultant for Edwards Lifesciences, Medtronic, and Corcym. All other authors reported no conflicts of interest.

The *Journal* policy requires editors and reviewers to disclose conflicts of interest and to decline handling or reviewing manuscripts for which they may have a conflict of interest. The editors and reviewers of this article have no conflicts of interest.
