# Supplementary material for: Aortic valve repair versus biological replacement for aortic regurgitation with or without ascending aortic aneurysm: Heart Valve Society Aortic Valve Database Study
Source: JTCVS Open. 2026 Aug 26;33:102085. doi: 10.1016/j.xjon.2026.102085 (PMC13711288; doi:10.1016/j.xjon.2026.102085)
Supplement: Supplementary file 1 [file mmc1.docx]

Supplementary Online Material

**Contents**

**Supplement 1.** Recorded variables with their corresponding percentages of missing

*Legend: NYHA, New York Heart Association; CCS, Canadian Cardiovascular Society; LVEF, Left Ventricular Ejection Fraction; COPD, chronic obstructive pulmonary disease; IDDM, insulin-dependent diabetes mellitus; NIDDM, noninsulin-dependent diabetes mellitus; MI; myocardial infarction*

**Supplement 2**. Supplementary methods, details regarding multiple imputation of missing baseline data

**Supplement 3.** Supplementary methods, details regarding propensity score matching and inverse probability treatment weighting.

**Supplement 4.** Distribution of year of primary surgery of all patients.

**Supplement 5.** Density plots of the propensity score in the unmatched and matched cohorts.

**Supplement 6.** Unmatched and matched pre-surgery echocardiographic measurements, aortic valve morphology and surgical details total cohort and stratified for surgical technique (aortic valve repair vs. biological aortic valve replacement).

**Supplement 7.** Unmatched and matched time-to event outcomes: (A) late aortic valve related reintervention after surgery, (B) endocarditis, (C) severe valve dysfunction, (D) moderate valve dysfunction (E) aortic complication, (F) other cardiac reoperation, (G) pacemaker implantation,

Every figure: unmatched patients who had undergone aortic valve repair or biological aortic valve replacement (A) matched patients who had undergone aortic valve repair or biological aortic valve replacement (B). Every figure contains the corresponding 95%CI.

**Supplement 8**. Weighted-Cox-Hazard Model for the outcomes after surgery. IPTW weighting of aortic valve repair to biological valve replacement.

**Supplement 1.**

|  | Missing (n) | Total patients | % Missing | Imputed |
| --- | --- | --- | --- | --- |
| Sex | 126 | 7543 | 1.7% | Yes |
| Age | 21 | 7543 | 0.3% | Yes |
| Reason for referral | 0 | 7543 | 0% | Yes |
| Location of aneurysm | 4585 | 7543 | 60.8% | No |
| Aortic diameter | 4480 | 7543 | 59.4% | No |
| Prosthesis failure | 7503 | 7543 | 99.5% | No |
| Other aetiology | 7502 | 7543 | 99.5% | No |
| Height | 77 | 7543 | 1.0% | Yes |
| Weight | 83 | 7543 | 1.1% | Yes |
| NYHA class | 475 | 7543 | 6.3% | Yes |
| CCS class of IV | 4730 | 7543 | 62.7% | No |
| Heart rhythm | 284 | 7543 | 3.8% | Yes |
| Previous surgery | 2571 | 7543 | 34.1% | Yes |
| Previous LVEF | 981 | 7543 | 13.0% | Yes |
| Hypertension | 2579 | 7543 | 34.2% | Yes |
| COPD | 2573 | 7543 | 34.1% | Yes |
| IDDM | 2622 | 7543 | 34.8% | Yes |
| NIDDM | 4414 | 7543 | 58.5% | Yes |
| Diabetes treatment | 7383 | 7543 | 97.9% | No |
| Creatinine | 391 | 7543 | 5.2% | Yes |
| Dialysis | 2683 | 7543 | 35.7% | Yes |
| Poor mobility | 2678 | 7543 | 35.5% | Yes |
| Extra arteriopathy | 2680 | 7543 | 35.5% | Yes |
| Recent MI | 2662 | 7543 | 35.3% | Yes |
| Critical state | 2703 | 7543 | 35.8% | Yes |
| Pulmonary hypertension | 475 | 7543 | 6.3% | Yes |
| Connective tissue disease | 3043 | 7543 | 40.3% | Yes |
| Number of aortic valve leaflets | 31 | 7543 | 0.4% | Yes |
| Aortic regurgitation grade | 755 | 7543 | 10.0% | Yes |

**Supplement 2.**

Imputations were done on all patients who underwent aortic valve repair or aortic valve replacement (biological prosthesis or mechanical prosthesis) for aortic regurgitation and ascending aortic aneurysm included Heart Valve Society Aortic Valve Database Study.

Selected baseline variables with <35% missingness were imputed, however some exceptions were made (Supplement 1). Imputation was performed in accordance with the considerations previously described.(1) Missing values of <40% was found in 23 out of the 29 imputed data. Nonetheless. 12 out of the 29 imputed data had <15% missing values. The imputations were made based on other baseline variables and the survival outcome by means of the Nelson-Aalen estimator. Correlation was tested with Spearman’s coefficient. wherein a correlation of <-0.40 or >0.40 was considered strong. In case of strongly correlated values. the most clinically relevant one was chosen to be a predictor. Five imputed datasets were generated with this method, using 25 interactions each. Convergence was checked by strip plots and density plots. and no major differences were noted between imputed data and observed data. Propensity score matching and subsequent time-to-event analyses were performed on each dataset separately and log-rank tests and Gray’s tests were pooled according to Rubin’s Rules using D2 statistics.(2)

Inverse probability treatment weighting and subsequent weighted cox hazard models were performed on each dataset separately as well and pooled according to Rubins Rules.

**Supplement 3.**

Propensity score matching was performed to balance baseline differences in pairwise-comparisons (aortic valve repair (REPAIR) vs. biological aortic valve replacement (bAVR)), as the main interest was the treatment effect of REPAIR in patient typically treated with bAVR. A multivariable logistic regression model was developed to estimate the propensity scores for every imputed dataset. The type of surgery was the dependent variable (REPAIR/bAVR). The REPAIR group was matched in a 2:1 ratio to bAVR using a calliper size of 0.2 and a nearest neighbour matching strategy. The models included age at surgery, sex, and concurrent surgeries (aortic root replacement, tubular aortic replacement, proximal arch replacement, coronary artery bypass grafting, mitral and/or tricuspid valve surgery, and other surgeries) as independent variables. Other baseline variables were added until all clinically relevant variables had a satisfactory balance with a standardized mean difference of <0.1 in all 5 matched cohorts for various patient characteristics which included: sex, age at surgery, referral reason, height, weight, NYHA class, heart rate, previous surgery, preoperative LVEF, hypertension, COPD, diabetes, creatinine level, dialysis, poor mobility, extracardiac arteriopathy, recent myocardial infarction, critical preoperative state, pulmonary hypertension, connective tissue disease, elective surgery status, aortic valve leaflet morphology, echocardiographic aortic insufficiency grade, isolated versus combined aortic pathology (tubular and root involvement), and concomitant procedures (CABG, aortic surgery, mitral valve surgery, MAZE procedure, and others). Time-to-event outcomes were performed on every imputed dataset. Post-match survival and MAVRE were calculated and visualized using the Kaplan-Meier method and compared (REPAIR to bAVR) using the log-rank test. All other post-match outcomes/adverse events were presented as cumulative incidence functions and analysed in the presence of death as a competing risk using the Aalen-Johanssen. Comparisons between the surgical groups were done using the log-rank test and Gray’s test, both pooled according to Rubin’s Rules using D2 statistics. Estimates of time-to-event outcomes are shown of the first imputed datasets.

To gain insights in the average treatment effect the inverse propensity treatment weighting (IPTW) method was applied.(3) Weights were assessed based on the inverse of the propensity score. IPTW analyses were performed weighting REPAIR to bAVR. The IPTW strategy was comparable to the propensity score matching strategy. Independent variables included age at surgery, sex and concurrent surgeries. Other clinically relevant baseline variables were added until all variables of interest had satisfactory balance with a standardized mean difference of <0.1. Time-to-event analysis for were performed by performing weighted-Cox- proportional hazard models for all five-imputed weighted cohorts. Cox models were pooled according to Rubins Rules.

**Supplement 4.**

**Supplement 5.**

**Supplement 6.**

|  | Before matching | | | | After matching | | | |
| --- | --- | --- | --- | --- | --- | --- | --- | --- |
|  | **Overall (n=7.318)** | **Aortic valve repair (n=6.773)** | **Biological valve replacement (n=545)** | **P-value** | **Overall (n=1.536)** | **Aortic valve repair (n=1006)** | **Biological valve replacement (n=530)** | **SMD** |
| Dilated Left Ventricular End-Diastolic Diameter (mm) | 59.0 [53.0. 65.0] | 59.0 [53.0. 65.0] | 60.0 [52.0. 66.0] | 0.990 | 59.0 [53.0. 65.0] | 59.0 [53.0. 65.0] | 60.0 [52.0. 66.0] | 0.011* |
| Dilated Left Ventricular End-Systolic Diameter (mm) | 40.0 [35.0. 46.0] | 40.0 [35.0. 46.0] | 40.0 [34.0. 45.0] | 0.148 | 40.0 [34.0. 45.0] | 40.0 [35.0. 46.0] | 40.0 [34.0. 45.0] | 0.066* |
| Pre-operative aortic valve mean gradient (mmHg) | 7.0 [4.0. 10.0] | 7.0 [4.0. 10.0] | 7.0 [5.0. 11.0] | 0.045 | 7.0 [5.0. 10.0] | 6.0 [4.0. 10.0] | 7.0 [5.0. 11.0] | 0.095* |
| Pre-operative aortic regurgitation grade (n) |  |  |  |  |  |  |  |  |
| None (n) | 385 (5.3) | 352 (5.2) | 33 (6.1) | 0.350 | 84 (5.5) | 51 (5.1) | 33 (6.2) | 0.025 |
| Mild (n) | 955 (13.1) | 894 (13.2) | 61 (11.2) | 0.301 | 201 (13.1) | 142 (14.1) | 59 (11.1) | 0.025 |
| Mild to moderate (n) | 1189 (16.2) | 1104 (16.3) | 85 (15.6) | 0.939 | 236 (15.4) | 152 (15.1) | 84 (15.8) | 0.025 |
| Moderate to severe (n) | 2529 (34.6) | 2355 (34.8) | 174 (31.9) | 0.397 | 511 (33.3) | 342 (34.0) | 169 (31.9) | 0.025 |
| Severe (n) | 1528 (20.9) | 1404 (20.7) | 124 (22.8) | 0.148 | 324 (21.1) | 204 (20.3) | 120 (22.6) | 0.025 |
| Aortic diameter (n) |  |  |  |  |  |  |  |  |
| Aortic annulus diameter (mm) | 27.0 [24.0. 29.0] | 27.0 [24.0. 29.0] | 26.0 [24.0. 30.0] | 0.897 | 26.0 [24.0. 29.0] | 26.0 [24.0. 29.0] | 26.0 [24.0. 30.0] | 0.032* |
| Aortic sinus diameter (mm) | 43.0 [38.0. 49.0] | 43.0 [38.0. 48.0] | 43.0 [38.0. 49.0] | 0.760 | 43.0 [38.0. 49.0] | 43.0 [38.0. 49.0] | 43.0 [38.0. 49.0] | 0.010* |
| Aortic STJ diameter (mm) | 39.0 [33.0. 45.0] | 39.0 [34.0. 44.0] | 39.0 [33.0. 45.0] | 0.879 | 39.0 [33.0. 45.0] | 39.0 [34.0. 45.0] | 39.0 [33.0. 45.0] | 0.020* |
| Tubular ascending aortic diameter (mm) | 45.0 [38.0. 51.0] | 45.0 [38.0. 51.0] | 46.0 [38.0. 52.0] | 0.790 | 45.0 [38.0. 51.0] | 44.0 [38.0. 51.0] | 46.0 [38.0. 52.0] | 0.090* |
| Aortic valve morphology |  |  |  |  |  |  |  |  |
| Tricuspid aortic valve (n) | 3956 (54.1) | 3601 (53.2) | 355 (65.1) | **<0.001** | 1009 (65.7) | 666 (66.2) | 343 (64.7) | 0.021 |
| Bicuspid aortic valve (n) | 3055 (41.7) | 2906 (42.9) | 149 (27.3) | **<0.001** | 464 (30.2) | 317 (31.5) | 147 (27.7) | 0.021 |
| Unicuspid aortic valve (n) | 239 (3.3) | 220 (3.2) | 19 (3.5) | 0.749 | 24 (1.6) | 6 (0.6) | 18 (3.4) | 0.021 |
| Quadricuspid aortic valve (n) | 41 (0.6) | 38 (0.6) | 3 (0.6) | 1.000 | 18 (1.2) | 15 (1.5) | 3 (0.6) | 0.021 |
| Operative characteristics |  |  |  |  |  |  |  |  |
| Repair type (n) |  |  |  |  |  |  |  |  |
| Isolated aortic valve repair (n) | 1887 (25.8) | 1887 (27.9) | - | - | 366 (23.8) | 366 (36.4) | 0 (0.0) | 0.044 |
| Tubular aortic replacement + aortic valve repair (n) | 1204 (16.5) | 1204 (17.8) | - | - | 251 (16.3) | 251 (25.0) | 0 (0.0) | -0.022 |
| Partial root replacement + aortic valve repair (n) | 177 (2.4) | 177 (2.6) | - | - | 33 (2.1) | 33 (3.3) | 0 (0.0) | -0.026 |
| Valve sparing root + aortic valve repair (n) | 3466 (47.4) | 3466 (51.2) | - | - | 356 (23.2) | 356 (35.4) | 0 (0.0) | -0.026 |
| David (n) | 1074 (14.7) | 1074 (15.9) | - | - | 134 (8.7) | 134 (13.3) | 0 (0.0) | -0.026 |
| Yacoub (n) | 2384 (32.6) | 2384 (35.2) | - | - | 221 (14.4) | 221 (22.0) | 0 (0.0) | -0.026 |
| Patch |  |  |  |  |  |  |  |  |
| Patch used (n) | 521 (7.1) | 521 (7.7) | - | - | 62 (4.0) | 62 (6.2) | - | - |
| Patch for cusp belly (n) | 101 (1.4) | 101 (1.5) | - | - | 16 (1.0) | 16 (1.6) | - | - |
| Patch for commissural reconstruction (n) | 194 (2.7) | 194 (2.9) | - | - | 25 (1.6) | 25 (2.5) | - | - |
| Patch for cusp extension (n) | 255 (3.5) | 255 (3.8) | - | - | 28 (1.8) | 28 (2.8) | - | - |
| Annuloplasty |  |  |  |  |  |  |  |  |
| Aortic annuloplasty (n) | 4135 (56.5) | 4134 (61.0) | 1 (0.2) | **<0.001** | 625 (40.7) | 624 (62.0) | 1 (0.2) | - |
| External ring (n) | 1767 (24.1) | 1766 (26.1) | 1 (0.2) | 1.000 | 287 (18.7) | 286 (28.4) | 1 (0.2) | - |
| Internal ring (n) | 43 (0.6) | 43 (0.6) | - | - | 6 (0.4) | 6 (0.6) | - | - |
| Suture annuloplasty (n) | 1686 (23.0) | 1686 (24.9) | - | - | 127 (8.3) | 127 (12.6) | - | - |
| STJ ring (n) | 270 (3.7) | 270 (4.0) | - | - | 60 (3.9) | 60 (6.0) | - | - |
| Valve replacement (n) |  |  |  |  |  |  |  |  |
| Isolated valve replacement (n) | 223 (3.0) | - | 223 (40.9) | - | 209 (13.6) | 0 (0.0) | 209 (39.4) | 0.044 |
| Root replacement + aortic valve replacement (n) | 193 (2.6) | - | 193 (35.4) | - | 192 (12.5) | 0 (0.0) | 192 (36.2) | -0.026 |
| Tubular aortic replacement+ aortic valve replacement (n) | 129 (1.8) | - | 129 (23.7) | - | 129 (8.4) | 0 (0.0) | 129 (24.3) | -0.022 |
| Additional procedures performed (n) |  |  |  |  |  |  |  |  |
| CABG (n) | 649 (8.9) | 573 (8.5) | 76 (13.9) | 0.001 | 210 (13.7) | 139 (13.8) | 71 (13.4) | -0.011 |
| Aortic (hemi) arch (n) | 735 (10.0) | 701 (10.3) | 34 (6.2) | <0.001 | 108 (7.0) | 74 (7.4) | 34 (6.4) | -0.027 |
| Mitral valve (n) | 519 (7.1) | 476 (7.0) | 43 (7.9) | 1.000 | 130 (8.5) | 87 (8.6) | 43 (8.1) | -0.028 |
| MAZE (n) | 349 (4.8) | 323 (4.8) | 26 (4.8) | 0.638 | 87 (5.7) | 61 (6.1) | 26 (4.9) | -0.058 |
| Other procedures (n) | 2327 (31.8) | 2262 (33.4) | 65 (11.9) | <0.001 | 184 (12.0) | 119 (11.8) | 65 (12.3) | 0.023 |

*SMD of unimputed data. Percentages were calculated including missing data.

LVEF = Left Ventricular Ejection Fraction; STJ = sinotubular junction; CABG = coronary artery bypass graft.

**Supplement 7.**

(A) Late aortic valve related reintervention

(B) Infective endocarditis

(C) Severe valve dysfunction

(D) Moderate valve dysfunction

(E) Aortic complication

(F) Other cardiac reoperation

(G) Pacemaker implantation

**Supplement 8.**

| Aortic valve repair vs Biological aortic valve replacement IPTW | | | |
| --- | --- | --- | --- |
| Outcome | **Variable** | **Hazard ratio (95% CI)** | **P-value** |
| Mortality | Surgical group (biological aortic valve replacement) | 2.2 (1.3-3.7) | **0.002** |
| MAVRE | Surgical group (biological aortic valve replacement) | 2.8 (1.6-4.9) | **<0.001** |
| Late aortic valve reintervention | Surgical group (biological aortic valve replacement) | 1.0 (0.3- 3.3) | 0.949 |
| Other cardiac reintervention | Surgical group (biological aortic valve replacement) | 1.3 (0.5- 3.1) | 0.623 |
| Endocarditis | Surgical group (biological aortic valve replacement) | 8.5 (2.2- 33.0) | **0.002** |
| Valve thrombosis | Surgical group (biological aortic valve replacement) | 1.4 (0.6-3.4) | 0.401 |
| Aortic complication | Surgical group (biological aortic valve replacement) | 0.4 (0.1- 1.7) | 0.234 |
| Pacemaker | Surgical group (biological aortic valve replacement) | 1.4 (0.6- 3.2) | 0.507 |
| Moderate valve dysfunction | Surgical group (biological aortic valve replacement) | 0.6 (0.3-1.4) | 0.269 |
| Severe valve dysfunction | Surgical group (biological aortic valve replacement) | 1.5 (0.7- 3.1) | 0.319 |

**REFERENCES**

1.	Papageorgiou G. Grant SW. Takkenberg JJM. Mokhles MM. Statistical primer: how to deal with missing data in scientific research? Interact Cardiovasc Thorac Surg 2018;27:153-158.

2.	Rubin DB. Schenker N. Multiple imputation in health-care databases: an overview and some applications. Stat Med 1991;10:585-98.

3.	Allan V. Ramagopalan SV. Mardekian J et al. Propensity score matching and inverse probability of treatment weighting to address confounding by indication in comparative effectiveness research of oral anticoagulants. J Comp Eff Res 2020;9:603-614.
